# Synthetic Pathways and the Therapeutic Potential of Quercetin and Curcumin

**DOI:** 10.3390/ijms232214413

**Published:** 2022-11-20

**Authors:** Aseel Ali Hasan, Victor Tatarskiy, Elena Kalinina

**Affiliations:** 1 T.T. Berezov Department of Biochemistry, Peoples’ Friendship University of Russia (RUDN University), 6 Miklukho-Maklaya Street, 117198 Moscow, Russia; 2 Laboratory of Molecular Oncobiology, Institute of Gene Biology, Russian Academy of Sciences, 34/5 Vavilov St., 119334 Moscow, Russia

**Keywords:** polyphenols, quercetin, curcumin, anti-oxidant and pro-oxidant effects, anti-cancer treatment

## Abstract

Polyphenols are considered popular ingredients in the pharmaceutical and medical fields due to their preventive and therapeutic properties. However, the potential effects and mechanisms of action of individual polyphenols remain largely unknown. Herein, we analyzed recent data on the synthetic pathways, features, and similarity of the properties of quercetin, as the most famous flavonoid, and curcumin, a representative of curcuminoids that despite their anti-oxidant activity, also have a pro-oxidant effect, depending on the concentration and the cellular environment. This review focuses on an analysis of their anti-cancer efficacy against various cancer cell lines via cell cycle arrest (regulation of p53/p21 and CDK/cyclins) and by triggering the mitochondrial intrinsic (Bcl-2/Bax/caspase 9) apoptotic pathway, as well as through the modulation of the signaling pathways (PI3K/Akt, Wnt/β-catenin, JAK/STAT, MAPK, p53, and NF-ĸB) and their influence on the non-coding RNAs involved in angiogenesis, invasion, migration, and metastasis. The therapeutic potential of quercetin and curcumin is discussed not only on the basis of their anti-cancer effects, but also with regard to their anti-diabetic, anti-obesity, anti-inflammatory, and anti-bacterial actions.

## 1. Introduction

Polyphenols comprise a widely distributed group of natural bioactive phytochemicals [1]. Structurally, these agents are represented by aromatic rings with hydroxyl groups. Besides the anti-oxidant activity, polyphenols have shown additional beneficial effects on human health [2]. Considerable evidence from epidemiological studies has demonstrated that dietary polyphenol intake is associated with protection against the development of several cancer types and against acute and chronic disorders, including cardiovascular and neurodegenerative diseases, osteoporosis, and diabetes mellitus [3,4]. Oxidative stress has been implicated in the development of these diseases [4]. Therefore, polyphenols play important roles in controlling the overproduction of reactive oxygen species (ROS) and reducing oxidative stress [5] via modulation of the redox signaling pathways [6].

Despite their anti-oxidant activity, polyphenols also evoke a pro-oxidant effect, which has been associated with their pro-apoptotic effect in cancer cells. Thus, depending on the concentration and cellular context, polyphenolic compounds can act as anti-oxidants or pro-oxidants [7]. Different classes belonging to this group are classified based on their structures; polyphenols essentially describe phenolic acids, stilbenes, flavonoids, lignans, and curcuminoids [8].

In this work, we critically review the current knowledge of the most important properties of two common polyphenol compounds, namely, quercetin, as an example of a flavonoid group; and curcumin, as an example of a curcuminoid group, and analyze the experimental evidence supporting their anti-diabetic, anti-obesity, anti-inflammatory, anti-viral, and antimicrobial actions. We also examine the anti-cancer mechanisms of quercetin and curcumin in human cell lines, as well as the combinatorial effects that have been validated clinically or experimentally.

## 2. Structure and Synthetic Pathways of Flavonoids and Curcuminoids

**Figure 1:**
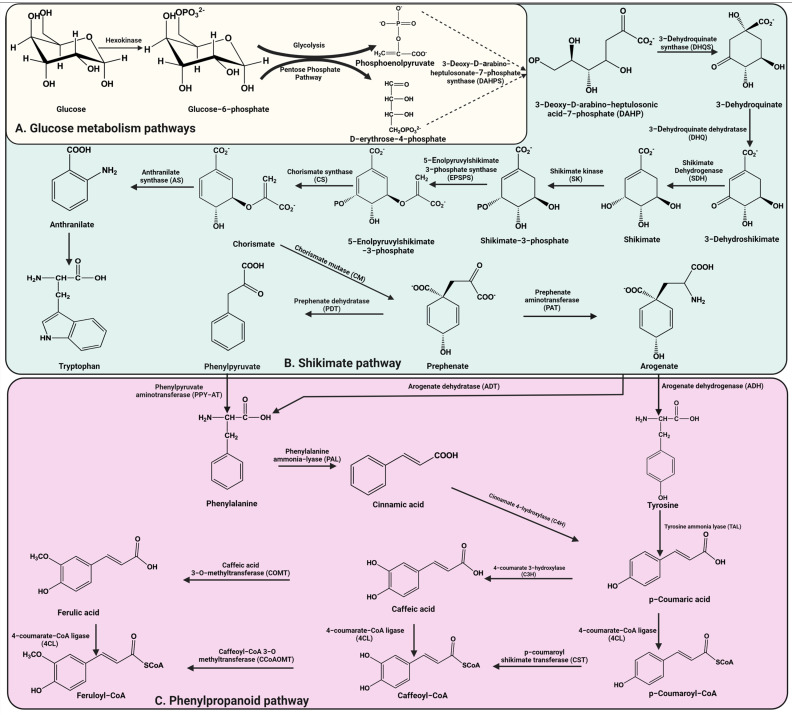
Biosynthesis pathways of polyphenol precursors.

In plants, polyphenols are secondary metabolites that play important roles in the defense against UV radiation and pathogens [3] and are synthesized through two metabolic pathways: the shikimate pathway and the phenylpropanoid pathway (Figure 1) [9].

The shikimate pathway includes seven sequential enzymatic reactions, starting with the condensation of phosphoenolpyruvate and D-erythrose-4-phosphate derived from the glycolytic and the pentose phosphate pathways, respectively (Figure 1A), to generate the final product, chorismite (Figure 1B) [10]. The aromatic amino acids phenylalanine and tyrosine formed through the shikimate pathway serve as precursors for the synthesis of phenylpropanoids (Figure 1C) [11]. Plants synthesize phenylalanine predominantly in plastids via the arogenate pathway, but knowledge about the biosynthesis of phenylalanine via the phenylpyruvate pathway is still unclear. Newly published research has provided evidence of phenylalanine biosynthesis via the phenylpyruvate pathway localized in the cytosol [12].

### 2.1. Flavonoids

Flavonoids represent the most ubiquitous polyphenolic compounds [13] and are responsible for flavor, color, and pharmacological activities [14]. Flavonoids also comprise a large group of polyhydroxy aromatic core compounds distributed widely in the plant kingdom [15]. More than 8000 flavonoid-derived compounds have been documented [16].

**Figure 2:**
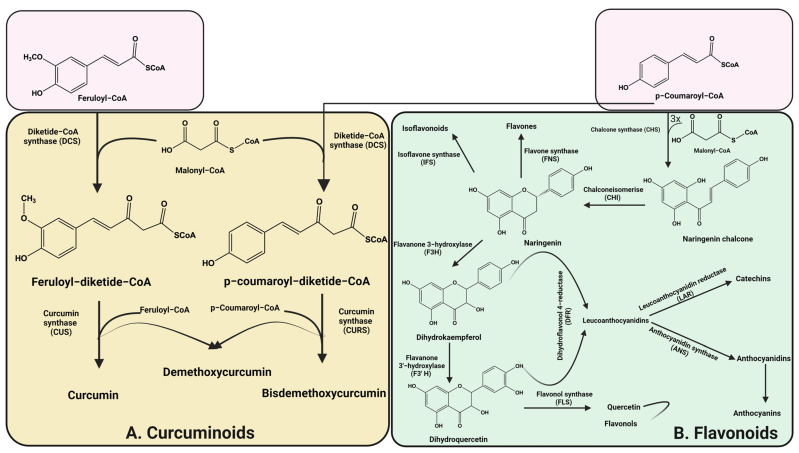
Biosynthesis pathways of flavonoids and curcuminoids.

**Figure 3:**
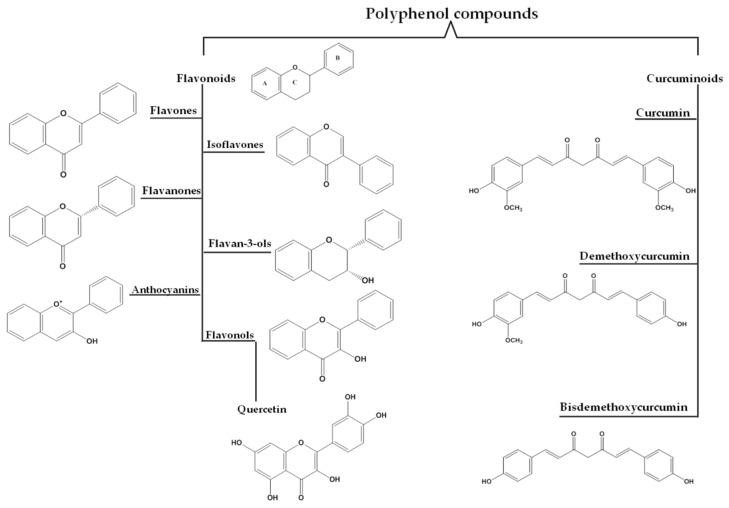
Structure of flavonoids and curcuminoids, major classes of polyphenols.

Flavonoid biosynthesis (Figure 1 and Figure 2B) begins with the aromatic amino acid through the phenylpropanoid pathway followed by a complicated series of enzymes, including synthases (isoflavone synthase (IFS), flavone synthase (FNS), flavonol synthase (FLS), anthocyanidin synthase (ANS), hydroxylases (flavanone 3-hydroxylase, F3H; flavanone 3′-hydroxylase, F3′H), reductase (leucoanthocyanidin reductase, LAR), and isomerase (chalconeisomerase, CHI)), which play critical roles in the modification of flavonoids and in the production of different classes of the flavonoid skeleton in higher plant species (Figure 2B) [17]. The six major subclasses of flavonoids, including flavones, isoflavones, flavanones, flavanols, flavan-3-ols (catechins), and anthocyanins, have a main backbone structure that is composed of two benzene rings (A and B) linked via a 3-carbon heterocyclic ring (ring C) [18]. The basic flavonoid structure and main subclasses are illustrated in Figure 3.

This field of research has witnessed a continuously growing interest in flavonoid derivatives in recent decades due to their anti-inflammatory and anti-cancer effects, anti-oxidative activity, and free radical scavenging capacity [19,20]. Among the flavonoid compounds, the most significant flavonol is quercetin [21].

#### Quercetin

Quercetin, as the most abundant flavonoid (Figure 3), is widespread in nature and can be found in plants, fruits, and vegetables, such as onion, cabbage, tea, apples, nuts, and berries [21]. The name quercetin is derived from the Latin word “quercetum”, which means oak forest [22]. The International Union of Pure and Applied Chemistry (IUPAC) nomenclature for quercetin is 3,3′,4′,5,7-pentahydroxyflavone. It is also known by its synonym 3,3′,4′,5,7-pentahydroxy-2-phenylchromen-4-one [23].

Chemically, quercetin occurs as an aglycone, lacking an attached sugar moiety, or as a glycoside (with linked sugars such as glucose, rhamnose, or rutinose). A functional glycosyl group attached to the quercetin skeleton may cause changes in solubility, absorption, and in vivo effects. Compared with quercetin aglycone, the conjugated form of quercetin glycoside is more completely absorbed [23,24,25].

Both in vitro and in vivo experiments showed that quercetin has many effects, such as anti-inflammatory and anti-oxidant effects [26]. Since quercetin does not harm healthy cells and is cytotoxic to cancer cells, it is therefore considered an anti-cancer through its ability to inhibit various types of cancers (including breast, lung, nasopharyngeal, kidney, colorectal, prostate, pancreatic, and ovarian cancers [27]), by its anti-inflammatory, pro-oxidative, anti-proliferation, apoptosis-promoting, and cell cycle arrest induction activities by inhibiting angiogenesis and metastasis progression and by affecting autophagy [26,27], which are discussed in detail below.

### 2.2. Curcuminoids

Curcuminoids are natural active polyphenolic compounds derived from turmeric. Curcuminoids are commonly used as a spice, pigment, or food additive and also have extensive biological activities, such as anti-oxidant, neuroprotective, anti-inflammatory, anti-cancer, antimicrobial, anti-viral, anti-angiogenic, anti-proliferative, anti-immunomodulatory, and anti-diabetic properties [28]. Curcuminoid biosynthesis (Figure 2A) in the herb *C. longa* includes the activities of two enzymes of type III polyketide synthases (PKSs): diketide-CoA synthase (DCS) and curcumin synthase (CURS). The starter substrates, p-coumaroyl-CoA or feruloyl-CoA, are synthesized from phenylalanine (Figure 1C) via the activity of the following enzymes: phenylalanine ammonia-lyase (PAL), cinnamate-4-hydroxylase (C4H), 4-coumarate-CoA ligase (4CL), p-coumaroyl shikimate transferase (CST), 4-coumarate-3-hydroxylase (C3H), and O-methyltransferases (OMT). DCS catalyzes the condensation of p-coumaroyl-CoA and feruloyl-CoA with malonyl-CoA to make diketide-CoAs, while CURS catalyzes the condensation between diketide-CoAs and starter substrates to form curcuminoids (Figure 2A) [29]. The pharmacological activity of turmeric has been basically attributed to three curcuminoids, namely, curcumin (CUR, which is the major curcuminoid), demethoxycurcumin (DMC), and bis-demethoxycurcumin (BDMC) *(*Figure 3) [30].

#### Curcumin

Curcumin (1,7-bis(4-hydroxy-3-methoxyphenyl)-1,6-heptadiene-3,5-dione; diferuloylmethane) is used as an everyday spice to provide a specific flavor and as an ingredient in traditional herbal medicine [31]. Curcumin’s biosynthetic route in turmeric includes the pathway in which DCS synthesizes feruloyldiketide-CoA, and CURS then converts diketide-CoA esters into a curcuminoid scaffold (Figure 2A). CURS itself was found to possess low activity for the synthesis of curcumin from feruloyl-CoA and malonyl-CoA; however, co-incubation of DCS and CURS was found to be very efficient in terms of curcumin yield [29].

The chemical structure of curcumin displays keto-enol tautomerism, with a predominant keto form in neutral and acidic solutions, whereas a stable enol form occurs in alkaline mediums [32]. Besides its low bioavailability and rapid chemical transformation due to metabolic enzymes in the gastrointestinal tract, the hydrophobic nature of curcumin after oral administration leads to a poor absorption rate by the gastrointestinal tract. Modern encapsulation technologies are the most effective means of protecting curcumin against chemical degradation, increasing its water dispersibility and improving its bioavailability [33].

Curcumin shows different beneficial pharmacological properties, especially anti-oxidant, anti-inflammatory, neuroprotective, anti-cancer, hepatoprotective, nephroprotective, and cardioprotective effects [34,35], with a particular emphasis on its anti-oxidant and anti-cancer properties.

## 3. Redox Properties and Biological Effects of Quercetin and Curcumin

### 3.1. Anti-Oxidant and Pro-Oxidant Properties

Oxidative stress is defined as an imbalance between pro- and anti-oxidation, triggering the oxidative mechanisms that have harmful effects [36,37].

The anti-oxidant mechanisms of polyphenols include the following: the suppression of ROS formation via the inhibition of the enzyme activities involved in their production, or via scavenging ROS directly by acting as hydrogen donors; the chelation of the metal ions that induce ROS production; and the inhibition of oxidative reactions by increasing the activity of anti-oxidant enzymes or the expression of anti-oxidant proteins and synergistically generating anti-oxidant effects with other substances [38]. The anti-oxidant activity of polyphenols is due to hydroxyl groups in their structure, which enable them to act as ROS scavengers [39].

Owing to its potent anti-oxidant capacity due to the presence of phenolic groups and a double bond in its structure, quercetin can eliminate free radicals and help maintain a stable redox state in cells by increasing anti-oxidant enzymes, such as superoxide dismutase (SOD), and catalase expressions, as well as the level of reduced glutathione (GSH) [40]. Furthermore, the free radical scavenging capacity of curcumin arises either from the phenolic OH group or from the CH_2_ group of the β-diketone (heptadienedione) moiety [41].

Molecular docking calculations indicate a stabilizing interaction between either quercetin or its main oxidized species and the kelch-like ECH-associated protein-1 (Keap1) domain in the nuclear factor erythroid 2-related factor 2 (Nrf2)-binding site. This leads to the release of the transcription factor Nrf2, the master regulator of anti-oxidant defenses [42], enhancing Nrf2 nuclear translocation and its binding activity [43], and promoting the expression of anti-oxidant response element (ARE)-dependent genes [42,43]. Quercetin can protect human granulosa cells from oxidative stress by inducing Nrf2 expression at both the gene and protein levels, which in turn induces the anti-oxidant thioredoxin (Trx) system. Quercetin not only significantly increases Trx expression but also suppresses thioredoxin-interacting protein (TXNIP), the internal suppressor of cellular Trx, leading to the inhibition of oxidative stress [43].

A meta-analysis revealed that pure curcumin, a metal chelator, directly removes ROS and regulates numerous enzymes. It has the potential to reduce the concentration of malondialdehyde (MDA) in serum and increase the total anti-oxidant potential [44]. Moreover, curcumin is an inhibitor of the ROS-generating enzymes cyclooxygenase and lipoxygenase [45].

Quercetin and curcumin are pivotal anti-oxidants that prevent the cell damage induced by oxidative stress. Oral administration of curcumin or quercetin demonstrated protective systemic effects against oxidative stress by decreasing the MDA level and stimulating anti-oxidant defense (ceruloplasmin and GSH levels) in adult male rats. In the serum, curcumin presented higher anti-oxidant effects compared with quercetin. In lungs, quercetin administration resulted in superior beneficial effects in the reduction of lipid peroxidation, but only temporarily [36]. However, the anti-oxidants quercetin and curcumin exhibit pro-oxidant activity depending on the specific set of conditions. Of particular importance are their dosage and redox conditions in the cell [37]. For instance, the low microencapsulation dose administration of quercetin (10 mg kg^−1^) gave rise to anti-oxidant and protective effects on interstitial cells of Cajal (ICC) and macrophages in the jejunum of diabetic rats. However, a high dose of quercetin (100 mg kg^−1^) aggravated the diabetic condition; furthermore, this treatment resulted in harmful effects on rats in the normoglycemic group, pointing to pro-oxidant activity [46]. The pro-oxidant capacity of quercetin is able to trigger apoptosis in several tumor cell lineages [47].

Density functional theory approaches have been used to elucidate the anti-oxidant and pro-oxidant properties of quercetin and its metal ion complexation capacity, as well as the anti-oxidant and pro-oxidant potential of metal ion–quercetin complexes. The pro-oxidant potential of quercetin has been evaluated via the reduction of [Quer-Cu(H_2_O)2]^2+^ complexes to [Quer-Cu(H_2_O)2]^+^ complexes with O_2_^–^ and ascorbate anion (Asc–). In this reaction, Cu(II) ion is reduced to Cu(I), which is involved in the Fenton-like reaction, producing HO^.^ radicals [48].

Curcumin irreversibly inhibits rat thioredoxin reductase (TrxR-1) activity through the alkylation of both residues in the catalytically active site (Cys^496^/Sec^497^) of the enzyme. This modification of TrxR-1 by curcumin, with a lack of Trx-reducing activity, showed a great increase in NADPH oxidase activity to produce ROS, shifting the enzyme function from anti-oxidant to pro-oxidant [49].

### 3.2. The Anti-Diabetic Effect

Diabetes mellitus is a chronic metabolic disorder and one of the five leading causes of death worldwide, characterized by persistent hyperglycemia associated with oxidative stress, along with carbohydrate, protein, and lipid metabolism abnormalities [50]. It may be caused by altered insulin secretion, resistance to the peripheral effects of insulin, or both [51,52], as well as alterations in glucose and lipid metabolism-regulating enzymes [50]. Chronic hyperglycemia in diabetes is associated with an increased risk of long-term problems, most prominent of which are microvascular (retinopathy, nephropathy, and neuropathy) and macrovascular (coronary artery disease, peripheral artery disease, and cerebrovascular disease) complications [52]. Recently, emphasis has been placed on complementary and alternative treatments for diabetes focused on novel herbal and functional foods and their bioactive compounds [53].

Polyphenolic compounds may exert anti-diabetic effects via the inhibition of amylin aggregation and the modulation of oxidative stress, inflammation, and their beneficial effects on both β-cell survival and whole-body insulin sensitivity. These compounds can inhibit and destabilize self-assembly by amylin, which requires aromatic molecular structures that bind to misfolding monomers or oligomers, coupled with adjacent hydroxyl groups present on single phenyl rings [54].

Recent in vitro and in vivo results have shown the anti-diabetic potential of quercetin to maintain whole-body glucose homeostasis through its interaction with many molecular targets in the small intestine, pancreas, skeletal muscles, adipose tissue, and liver, and its ability to inhibit intestinal glucose absorption, insulin secretory, and insulin-sensitizing activities, as well as improve glucose utilization in peripheral tissues [55]. The consumption of low and high amounts of quercetin improved hyperglycemia, hypertriglyceridemia, and anti-oxidant status by reducing thiobarbituric acid reactive substances (TBARS) and elevating the anti-oxidant enzyme SOD, catalase, and glutathione peroxidase (GPx) in the liver of type 2 diabetic db/db mice [56].

Curcumin also has anti-diabetic effects. It showed potent inhibitory activity toward α- and β-glucosidase and α-amylase in a concentration-dependent manner [57]. Under a low dosage (300 mg/kg), it could decrease diabetic vascular inflammation by reducing leukocyte–endothelium interaction and inhibiting the expression of intercellular adhesion molecule 1 (ICAM-1) and pro-oxidant NADPH oxidase (NOX2) in a diabetic rat model [58]. Thus, curcumin’s anti-diabetic activity might be due to its capacity to suppress oxidative stress and inflammatory processes and to reduce fasting blood glucose, glycated hemoglobin (HbA1c), and body mass index [59].

Both curcumin and quercetin feeding modulated lysosomal enzymes (N-acetyl-β-d-glucosaminidase, β-d-glucuronidase, β-d-galactosidase, and acid phosphatase) in different tissues of streptozotocin-induced diabetic rats [60]. Due to the low bioavailability of curcumin, a combination of curcumin with quercetin as an enhancer presented significantly better therapeutic potential compared with curcumin alone. The oral administration of curcumin extract with piperine and quercetin (100 mg kg^−1^/day) significantly reduced plasma glucose by 28 days compared with streptozotocin- and nicotinamide-induced diabetic rats [61].

### 3.3. The Anti-Obesity Effect

Obesity is an unhealthy expansion and accumulation of adipose tissue to store excess energy intake, impairing physical and psychosocial health. It is associated with metabolic syndromes such as type 2 diabetes, insulin resistance and cardiovascular disease [62,63]. There are multiple strategies available for obesity management, which include bioactive polyphenols. These compounds can reduce energy and food intake, lipogenesis, and preadipocyte differentiation and proliferation, while stimulating energy expenditure by promoting lipolysis and β-oxidation at the same time [62].

Research has suggested that quercetin downregulates adipogenesis by suppressing the protein levels of the key adipogenic factors: CCAAT/enhancer-binding protein-β (C/EBPβ), CCAAT/enhancer-binding protein-α (C/EBPα), peroxisome proliferator-activated receptor-γ (PPARγ), and fatty acid-binding protein 4 (FABP4). It has also been found to inhibit the mitogen-activated protein kinase (MAPK) signaling factors—extracellular signal-regulated kinase (ERK1/2), c-Jun N-terminal kinase (JNK), and p38MAPK—as well as pro-inflammatory cytokine tumor necrosis factor (TNF)-α and chemokine monocyte chemoattractant protein-1 (MCP-1) in adipocytes and macrophages, along with inhibiting lipid accumulation and obesity-induced inflammation in 3T3-L1 preadipocytes, zebrafish, and mice models [64]. Moreover, quercetin has been shown to exert a direct and rapid downregulatory effect on the expression of sterol regulatory element-binding proteins (SREBP-1 and SREBP-2), transcriptional factors representing the main regulators of de novo fatty acid, and cholesterol synthesis in permeabilized C6 glioma cells. Quercetin also reduces carbohydrate response element-binding protein (ChREBP), which is involved in the regulation of lipogenic genes [65].

The effect of curcumin intake was correlated with a significant reduction in body mass index, weight, waist circumference, and leptin, and a significant increase in adiponectin levels among patients with metabolic syndrome and related disorders [66]. Bioactive curcumin exerts anti-adipogenic features through the inhibition of the expression levels of early adipogenic transcription factors, particularly Krüppel-like factor 5 (KLF5), C/EBPα, and PPARγ, during the early stage of adipocyte differentiation [67]. The curcumin-induced suppression of adipogenic differentiation is accompanied by the activation of Wnt/β-catenin signaling, the inhibition of MAPK (ERK, JNK, and p38) phosphorylation, and the expression of mature adipocyte marker aP2 in 3T3-L1 cells [68].

### 3.4. The Antimicrobial Effect

Antibiotic resistance is a growing public health concern that affects people, animals, the environment, and the economy. Polyphenols play an important role in a new strategy to prevent and treat bacterial infections. Furthermore, polyphenols act synergistically with various antibiotics, suggesting a promising alternative for use in therapeutic strategies against antibiotic resistance [69].

Flavonoids exert anti-bacterial properties due to the hydroxylation of C5, C7, C3′, and C4′, along with geranylation or prenylation at C6 [70]. Quercetin exhibits potent anti-microbial activity against different Gram-positive and Gram-negative bacteria, as well as fungi and viruses. The mechanisms of its anti-microbial activity include the disruption of cell membrane integrity, changes in membrane permeability, the inhibition of nucleic acid and protein synthesis, the inhibition of virulence factor expression, mitochondrial dysfunction, and the prevention of biofilm formation [71].

Curcumin blocks bacterial growth by inhibiting bacterial virulence factors and bacterial biofilm formation, and preventing bacterial adhesion to host receptors through the bacterial quorum-sensing regulation system [72].

### 3.5. The Anti-Inflammatory and Anti-Viral Action

Inflammation includes the secondary tissue alterations that occur during physical, chemical, mechanical, or infectious injuries. Inflammatory mechanisms are associated with the production of high levels of ROS, cytokines, and redox-dependent transcription factors [36].

Recent in vivo results showed that maternal quercetin consumption during lactation-modulated inflammatory responses and autophagy flux in the kidneys of high-fructose diet-fed adult female offspring by decreasing the amounts of macrophages as well as TNF-α and IL-6 mRNA levels [73,74]. Quercetin and its derivatives (quercetin-3-O-glucuronide, tamarixetin, isorhamnetin, isorhamnetin-3-O-glucoside, quercetin-3,4′-di-O-glucoside, and quercetin-3,5,7,3′,4′-pentamethylether) have shown significant anti-inflammatory and inhibition potential towards the synthesis of 12(S)-hydroxy(5Z,8E,10E)-heptadecatrienoic acid (12-HHT), thromboxane B2 (TXB2), prostaglandin E2 (PGE2), and 12(S)-hydroxy-(5Z,8Z,10E,14Z)-eicosatetraenoic acid (12-HETE)—inflammatory mediators derived from arachidonic acid metabolism, which are catalyzed by the inflammatory response enzymes cyclooxygenase-1 (COX-1) and 12-lipoxygenase (12-LOX) [75].

Curcumin also exhibits anti-inflammatory effects via the inhibition of macrophage infiltration and nuclear factor κB (NF-κB) activity induced by inflammatory agents in adipose tissue. In addition, curcumin reduces the expression of pro-inflammatory adipokines tumor necrosis factor-α (TNFα), monocyte chemoattractant protein-1 (MCP-1), and plasminogen activator inhibitor type-1 (PAI-1) [76]. Curcumin evoked anti-inflammatory activity in lipoteichoic acid-induced microglial cells by inhibiting TNF-α; prostaglandin E2 (PGE2); nitric oxide secretion; the phosphorylation of ERK, p38 MAPKs, and Akt; and the translocation of NF-κB, while it may also induce the expression of transcription factor Nrf2 and the anti-oxidant enzyme heme oxygenase 1 (HO-1) [77].

Severe acute respiratory syndrome coronavirus-2 (SARS-CoV-2, COVID-19), a pandemic beta coronavirus, has caused severe pneumonia, excessive inflammatory reactions, acute lung injury, and multiple organ dysfunction syndromes that are principally responsible for the death of patients. Quercetin, as an anti-inflammatory compound, may be an effective treatment for severe inflammation, one of the main life-threatening conditions in patients with COVID-19, by suppressing the production of pro-IL-1β and the NLR family pyrin domain containing 3 (NLRP3) inflammasome by affecting its regulators, such as TXNIP, SIRT1, and Nrf2 [78]. Current evidence shows that synergistic vitamin C and quercetin therapy exerts anti-viral and immunomodulatory properties for the prevention and treatment of COVID-19 [79].

Curcumin supplementation may offer an efficacious and safe option for improving COVID-19 disease in hospitalized patients through partial restoration of the pro-inflammatory/anti-inflammatory balance. Curcumin significantly decreases pro-inflammatory cytokines, such as IL1β and IL6, and increases anti-inflammatory cytokines, including IL-10, IL-35, and TGF-α [80].

### 3.6. The Anti-Cancer Effect

In addition to anti-oxidants, the polyphenolic compounds quercetin and curcumin are the most promising compounds with key regulatory roles in cancer development via the management of a wide range of cellular processes, such as differentiation, proliferation, apoptosis, the cell cycle, and responses to oxidative stress. The anti-cancer effects of these compounds are associated with their modification effect on the central elements of various signaling pathways, including MAPK, PI3K, Akt, and mTOR, along with their effect on the tumor-suppressing p53 protein and RAS oncoproteins [34,81,82]. A defect in the p53 signaling pathways is a critical factor in tumor initiation and progression [83]. Polyphenols regulate not only *p53* gene expression, but also post-translational modifications of p53, such as methylation, phosphorylation, acetylation, and ubiquitination, which, taken together, affect the functions of p53 in response to DNA damage, apoptosis control, cell cycle regulation, and senescence [81].

The anti-cancer effects of quercetin and curcumin have been reported in a series of recent studies and can be attributed to the mechanisms shown in Table 1.

For instance, in breast cancer MCF-7 cells, quercetin suppresses Twist, a major transcriptional activator of epithelial–mesenchymal transition, which in turn induces apoptosis through G1/S arrest by downregulating cyclin D1 and decreasing the phosphorylation of p38 MAPK [84]. In vitro, curcumin exhibited the potent growth inhibition of breast cancer cells MCF-7, T47D, and MDA-MB-415, with an IC_50_ at the micromolar range. Curcumin induced cell cycle arrest at the G2/M phase by decreasing CDC25 and CDC2 and increasing the p21 level, a key mechanism of proliferation inhibition. The PI3K/Akt/mTOR signaling pathway overexpressed in breast cancer leads to cell growth and tumor proliferation, while curcumin inhibited the phosphorylation and activation of the Akt/mTOR signaling pathway, decreased the expression of the anti-apoptotic protein BCL-2, and promoted Bax expression and caspase-3 protein cleavage [85]. In terms of cell viability, the curcumin analog B14 had a stronger inhibition rate than curcumin, with IC_50_ values of 16.85 μM and 42.01 μM for curcumin, and 8.84 μM and 8.33 μM for the B14 compound in MCF-7 and MDA-MB-231 cells, respectively. In this study, curcumin was less effective in inhibiting cell viability compared with the previous study on MCF-7 cells. The analog B14 reduced the expression of cyclin D1, cyclin E1, and cyclin-dependent kinase 2, resulting in cell cycle arrest at the G1 phase and activation of the mitochondrial apoptosis pathway [86].

Cyclin D1 expression was significantly decreased in quercetin-treated ovarian SKOV-3 cells, but not in cisplatin (CDDP)-resistant SKOV3/CDDP cells. This reduction in cyclin D1 expression could be linked to the G1/S phase alteration found in quercetin-treated cells. However, quercetin did not significantly affect cyclin B1 levels in both cell lines [87]. The levels of PI3K and phospho-Akt were decreased in curcumin-treated SKOV3 cells, which in turn increased caspase-3 and Bax levels. Curcumin also downregulated the levels of BCL-2 and coordinately inhibited anti-apoptotic effects [88].

In human HT-29 colorectal cancer cells, cell shrinkage, chromatin condensation, and nuclear collapse were observed after treatment with quercetin. Quercetin inhibits cell viability and induces apoptosis via Akt-CSN6-Myc signaling axis inhibition. Following quercetin treatment, p-Akt-Thr308 and CSN6 (a subunit of the constitutive photomorphogenesis 9 (COP9) signalosome (CSN)) protein expression levels were downregulated, leading to the modulation of CSN6 downstream gene expression; the levels of Myc and Bcl-2 were reduced, and p53 and Bax were increased [89]. Curcumin enhanced the efficacy of chemotherapy in colorectal cancer cells. Thus, a combination treatment regimen of 5-fluorouracil and curcumin in two colorectal cancer cell lines (HCT116 and HCT116+ch3, which was made MLH1-proficient by the stable transfection of chromosome 3 bearing the *hMLH1* gene) induced the expression or cleavage of pro-apoptotic proteins (caspase-8, -9, and -3; PARP; and Bax) and downregulated anti-apoptotic (BCL-XL) and proliferative (cyclin D1) proteins. While 5-fluorouracil activated the NF-κB/PI-3K/Src protein kinase signaling pathway, it was downregulated by curcumin via the inhibition of IκBα phosphorylation/activation [90].

In human lung cancer cells, quercetin showed an inhibitory effect on proliferation by inducing apoptosis via Bax, BCL-2, and caspase-3. In vivo, quercetin showed potential effects of reducing oxidative stress by increasing the SOD and GPx enzyme levels, and it improved the restoration of the damaged lung tissue caused by cyclophosphamide toxicity [91]. The H446 human small cell lung cancer (SCLC) cell line was most sensitive to curcumin compared with HCT116, Hela, MB231, PC-9, or A549 cells. Curcumin effectively promoted apoptosis, reduced the expression of BCL-2, and increased the expression of Bax and cytochrome c, which are involved in the regulation of H446 cell proliferation [92].

Quercetin was found to be significantly effective in inhibiting the proliferation of human bladder cancer 5637 and T24 cells in a dose-dependent manner. The caspase-3/7 activity in quercetin-treated cells increased the percentage of subG0/G1 cells and DNA fragmentation, indicating induced apoptotic cell death. Quercetin exerts dual roles in apoptosis and in protective autophagy. In quercetin-treated 5637 and T24 cells, LC3-II, the autophagic marker protein, was gradually increased concomitantly with autophagosome formation. The inhibition of autophagy by Baf1 and chloroquine, switched to the mode of cell death, was induced by quercetin to apoptosis. In addition, the data revealed that the decrease in cell viability and increase in LC3-II processing were attenuated in quercetin-treated cells that were pretreated with N-acetyl cystine, a ROS scavenger, suggesting that quercetin-induced cytotoxicity and autophagy were initiated by the generation of ROS [93]. The downregulation of Trop2, the human trophoblast cell surface antigen 2, by curcumin in bladder cell cancer lines suppressed cell proliferation and motility, decreased cyclin E1, and elevated p27 levels [94].

A novel stable hybrid molecule consisting of losartan, the selective antagonist of angiotensin II subtype 1 receptor as a ROS inhibitor, and quercetin, as the anti-oxidant, is able to regulate ROS levels through a dual mode of action. This quercetin–losartan hybrid can also modify the cell cycle, causing cell cycle arrest, the induction of cytotoxic effects, and a reduction in cancer cell proliferation and angiogenesis in primary *glioblastoma* multiforme (GBM) cultures, while quercetin alone inhibits angiogenesis by reducing segment and mesh formation [95].

In vitro, curcumin and its analogues—bisdemethoxycurcumin, demethoxycurcumin, and dimethoxycurcumin—increased early apoptosis, late apoptosis, and ROS production in LN229 and GBM8401 glioma cells. Curcumin and dimethoxycurcumin exhibited potential anti-cancer activities among curcumin analogues in human glioma cells that induced ROS production to increase autophagy, apoptosis, and suppressed cell viability [97].

**Figure 4:**
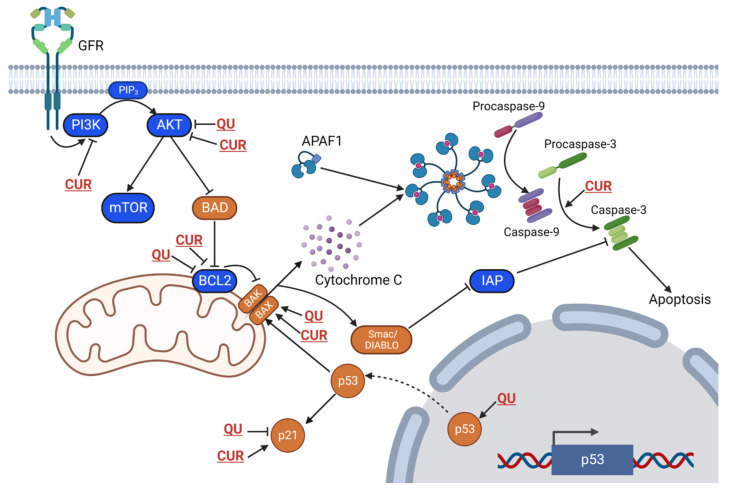
Signaling pathways modulated by quercetin and curcumin to induce tumor suppression and apoptosis. QU, quercetin; CUR, curcumin.

Quercetin induced cytotoxicity in three leukemic cell lines (Nalm6, K562, and CEM) in a dose-dependent manner, while in breast cancer T47D cells, quercetin has limited sensitivity with an IC_50_ value of 160 μM. During tumor regression, quercetin caused cell arrest at the S phase, and a dose-dependent increase in the sub-G1 population was observed. The anti-cancer mechanisms for both quercetin and curcumin include the activation pathways associated with apoptosis (Figure 4). Quercetin, as a DNA intercalator, induces DNA damage, which leads to the upregulation of p53, the downregulation of antiapoptotic protein BCL2, and the cleavage of MCL1, an apoptotic marker. Thus, the mitochondrial intrinsic pathway is stimulated by releasing cytochrome c and SMAC/DIABLO due to the loss of mitochondrial membrane potential. SMAC/DIABLO inhibits IAPs (inhibitors of apoptosis proteins), while cytochrome c release from mitochondria serves as a key step in the activation of downstream caspases. Cytochrome c takes part in the activation of an initiator, caspase-9, which in turn cleaves and activates caspase-3. Caspase-3 activates apoptosis via the fragmentation and degradation of cellular DNA and the activation of PARP1 [98]. Curcumin dose-dependently suppressed the phosphorylation of AKT, PRAS40, 4E-BP1, P70S6K, RAF-1, and p27 and regulated the cell cycle- and apoptosis-related proteins (cyclin D1, p21, Bcl2, cleaved caspase-3, and cleaved PARP), leading to cell cycle arrest and apoptosis in acute myeloid leukemia cell lines ML-2 and OCI-AML5 [99].

## 4. Non-Coding RNAs, A Novel Regulatory Network for Quercetin and Curcumin

Quercetin and curcumin exhibit anti-cancer activity and regulate cancer progression not only through the modification of multiple cellular signaling pathways, including the PI3K/Akt, Wnt/β-catenin, JAK/STAT, MAPK, p53, and NF-ĸB signaling pathways (Table 1, Figure 4), but also via the modification of non-coding RNAs (ncRNAs).

Since ncRNAs mediate cellular processes (for instance, chromatin remodeling, transcription, post-transcriptional modifications, and signal transduction), they act as biomarkers or key regulators of the cancer gene network and have functional roles in the onset and progression of malignancies [100]. Through various mechanisms, dysregulated ncRNAs may promote the hallmarks of cancer as either oncogenes or tumor suppressors, confirming the potential of ncRNAs for cancer treatment [101]. The functional relevance of ncRNAs is particularly evident for their two major classes—microRNAs (miRNAs) and long non-coding RNAs (lncRNAs) [102]—which play crucial roles with regard to the anti-cancer effects of polyphenols by targeting the main oncogenes or restoring tumor suppressor gene expression [103]. In comparison with well-utilized and studied miRNA, lncRNAs were more recently identified as important players against cancer [102]. Although thousands of lncRNA have been detected in eukaryotes, an understanding of the molecular mechanisms of lncRNA functions is limited due to an insufficient knowledge of their structure [104].

MiRNAs (endogenously expressed small non-coding RNA sequences, approximately 22 nucleotides) act as post-transcriptional regulators of gene expression, and their dysregulation may be associated with cancer transformation [105], including the cell cycle, apoptosis, cell migration, and epithelial–mesenchymal transition [102]. MiRNA biogenesis has the following multistep process: a primary microRNA (pri-miRNA) is transcribed via RNA polymerase II or III and then cleaved into pre-miRNA by Drosha with DGCR8. The resulting pre-miRNA is exported to the cytoplasm by exportin 5, where it is further processed by the Dicer enzyme to generate mature miRNA. Mature miRNA is integrated into the RNA-induced silencing complex (RISC). Finally, this complex binds to precise or partial complementarity mRNA sequences in the cytoplasm to mediate either mRNA degradation or the inhibition of its translation through its effects on translation initiation, elongation, and termination, as well as co-translational degradation [106]. The modulation of the function of miRNA by quercetin and curcumin is associated with either the downregulation of oncogenic miRNA or the upregulation of tumor-suppressive miRNA, thus targeting multiple genes and consequently affecting the various signaling pathways that control cell proliferation and apoptosis [107,108]. The following examples illustrate the impact of alterations in miRNA mechanisms caused by each of these compounds in the development and progression of diverse types of human cancers.

Computational analysis assumes that miR-200b-3p is a strong candidate for a cell-fate determinant in pancreatic ductal adenocarcinoma (PDA) cells by modulating Notch signaling. The induction expression of miR-200b-3p in PDA cells by quercetin may render the cancer stem cells less aggressive by a switch of the cell division mode from symmetric (self-renewing cancer stem cell division) to asymmetric division (differentiation to adipocytes, osteocytes, and chondrocytes), which is associated with reversing the elevated Notch levels with a simultaneous decrease in Numb protein levels [109]. 

The growth inhibitory effect of quercetin on MCF-7 cells may be mediated by a reduction in miR-21 expression and an increase in the expression of Maspin and PTEN, which are known targets of miR-21 and play an important role in the apoptosis pathway [110]. Furthermore, a combination of quercetin and hyperoside (3-O-galactoside of quercetin) also has an inhibitory effect on prostate tumor-associated miR-21 expression, which reduces the growth, invasion, and metastasis of prostate cancer PC3 cells. Thus, this combination increased the expression of programmed cell death protein 4 (PDCD4), a key target miR-21 protein, which was negatively regulated by miR-21 [111].

The overexpression of the miRNA344a-3p mimic combined with curcumin treatment enhanced the expression of apoptotic proteins, including procaspase-9 and cleaved caspase-3 in RT4 schwannoma cells [112]. Moreover, curcumin suppresses osteogenesis via the upregulated miR126a-3p that directly targets and inhibits human low-density lipoprotein receptor-related protein 6 (LRP6) expression and then suppresses WNT signaling activation, thus inhibiting the osteogenic differentiation of human adipose-derived mesenchymal stem cells (hMSCs). This finding suggests that the suppression of osteogenesis by the long-term use or large dosages of curcumin may lead to decreased bone mass and density, which may cause a potential threat to tumor metastasis [113].

It has been shown that curcumin exhibits inhibitory effects on the growth of PC-3 and DU-145 prostate cancer cells by enhancing miR-30a-5p and suppressing the PCNA clamp-associated factor (PCLAF) expressions that lead to an increase in the level of Bax and cleaved caspase-3 expression, and to a decrease in Bcl-2 and caspase-3 levels. Notably, the anti-cancer activity of curcumin was attenuated after the transfection of PCa cells with miR-30a-5p inhibitors, suggesting that miR-30a-5p was a downstream effector of curcumin, and the function of curcumin in inhibiting PCa progression was partly dependent on miR-30a-5p [114].

In U87 MG human glioblastoma cells, treatment with curcumin led to the upregulation of miR-146a, a negative regulator of NF-κB signaling, while combined curcumin and temozolomide treatment inhibited cell proliferation and induced apoptotic death compared with each alone. In U87 MG human glioblastoma cells, upregulation of miR-146a, a negative regulator of NF-κB signaling, mediates the sensitization of cells to temozolomide-induced apoptosis by curcumin [115].

## 5. Combinations with Antineoplastic Drugs

Despite the various anti-cancer strategies used in cancer treatment, chemotherapy remains a predominant therapeutic option. Multidrug resistance (MDR) refers to a phenomenon in which the cancer cells develop resistance to a broad spectrum of structurally and functionally unrelated anti-cancer drugs, one of the major hurdles to successful chemotherapy. Cancer cells mainly exhibit resistance to chemotherapeutic agents due to the overexpression of ATP-binding cassette (ABC) transporter proteins in the cell membrane, which extrude chemotherapeutic agents outside of cancer cells. The other MDR mechanisms can be explained by enhanced drug detoxification, DNA repair efficiency, defects in apoptosis regulation, and active cell survival signals. Natural products derived from plants, phytochemicals, have recently been defined as MDR-reversing agents, which act also as chemosensitizers in combination with chemotherapeutic agents to enhance their efficacy in cancer cells [116,117]. Growing evidence has proved that a combination of quercetin or curcumin with chemotherapeutic agents, as shown below, considerably enhances the antitumor potencies of doxorubicin (DOX) and cisplatin.

Co-treatment with quercetin and cisplatin synergistically inhibits proliferation, migration, and invasion and enhances apoptosis in HeLa and SiHa human cervical cancer cells by weakening matrix metallopeptidase 2 (MMP2), ezrin, P-glycoprotein, and methyltransferase-like 3 (METTL3) expressions [118]. Furthermore, the co-administration of quercetin and DOX can enhance the cytotoxicity of DOX toward MDA-MB-231/MDR1 cells, causing an increase in intracellular DOX accumulation through the downregulation of P-glycoprotein expression and the initiation of mitochondrial apoptotic pathways to facilitate DOX-induced apoptosis [119].

A combination treatment of curcumin and cisplatin improves the chemotherapeutic action of cisplatin against Hep2 human laryngeal squamous cancer cells, which could be related to the transient receptor potential melastatin 2 (TRPM2)-associated increase in excessive mitochondrial oxidative stress and Ca^2+^ influx [120]. A co-delivery system, CUR-PEI-K14/p53, for the simultaneous delivery of curcumin and p53 DNA significantly increases the sensitivity of SKOV-3/CDDP cells to cisplatin through the upregulation of p53 and Bax mRNA and the downregulation of *MDR1* mRNA [121]. Curcumin’s sensitizing effect on DOX has been related to the inhibition of the ATPase activity of ABC transporter family member 4 (ABCB4) in multidrug-resistant MCF-7/DOX and MDA-MB-231/DOX breast cancer cells [122]. It might also be related to the downregulation of the expression and function of Pgp and S100 calcium-binding protein A8 (S100A8), causing an intracellular calcium ion imbalance that resulted in increased endoplasmic reticulum stress and accelerating apoptosis in K562/DOX cells [123].

The synergistic treatment with curcumin and quercetin inhibited the cell proliferation associated with the loss of mitochondrial membrane potential (ΔΨm), the release of cytochrome c, a decrease in AKT and ERK phosphorylation in MGC803 human gastric cancer cells [124], and the downregulation of Wnt/β-catenin signaling pathway proteins, including DVL2, β-catenin, cyclin D1, Cox2, and Axin2, as well as the modulation of apoptotic pathways, including Bcl-2, caspase-3/7, and PARP cleavage in A375 melanoma cells [125].

## 6. Discussion and Conclusions

The development of safe, natural, and effective drugs with less toxicity and without side effects is necessary to treat the various incurable diseases causing death worldwide. Due to their unique chemical structures, polyphenols are considered to be some of the most important natural compounds.

Diverse studies at the molecular and protein levels have used quercetin and curcumin as model polyphenol compounds, providing strong evidence confirming the effective role of quercetin and curcumin in improving human health. Many of the effects attributed to these compounds are based on redox-related properties related to their anti-cancer, anti-diabetic, anti-obesity, anti-inflammatory, anti-viral, and anti-bacterial actions. In general, their anti-cancer mechanisms include the modulation of molecular events and signaling pathways, along with the interference of cell survival, proliferation, differentiation, angiogenesis, invasion, migration, and metastasis.

However, further research with adequate in vivo and in vitro investigations could lead to the development of more effective derivative compounds to further clarify the mechanisms presumably involved in the protective and curative roles of quercetin and curcumin as attractive therapeutic candidates in cancer and other diseases.

## Figures and Tables

**Table 1:** Cytotoxicity of quercetin and curcumin in cancer cell lines.

	Human Cell Line	Compound	IC_50_ (μM)	Cell Cycle Arrest	Target Action	Reference
Breast	MCF-7	Quercetin	37	G1	Induces apoptosis through suppression of cyclin D1, p21, Twist, and phosphor-p38MAPK	[84]
	MCF-7 T47D MDA MB 415	Curcumin	1.32 2.07 4.69	G2/M	Induces apoptosis by decreased CDC25 and CDC2, increased P21, inhibition of the phosphorylation of Akt/mTOR, decreased BCL2, and promoting Bax and cleavage caspase-3	[85]
	MCF-7 MDA-MB-231	Curcumin analog B14	8.84 8.33	G1	Reduces cyclin D1, cyclin E1, and CDK2. Activates the mitochondrial apoptosis pathway	[86]
Ovarian	SKOV-3	Quercetin	100	G2/M	Decreases cyclin D1	[87]
	SKOV-3	Curcumin	24.8	G2/M	Downregulates PI3K/Akt BCL-2, increases caspase-3 and Bax	[88]
Colon	HT-29	Quercetin	81.65	G0/G1	Reduces p-Akt, increases CSN6 protein degradation, which reduces Myc and BCL-2 and increases p53 and Bax	[89]
	HCT116 HCT116+ch3	Curcumin	20 5	S	Induces mitochondrial degeneration and cytochrome c release	[90]
Lung	A549	Quercetin	5.14	G2/M	Increases Bax, decreases BCL-2	[91]
	H446	Curcumin	~10	G2/M	Reduces BCL-2, increases Bax and cytochrome c	[92]
Bladder	5637 T24	Quercetin	47.91 67.26	Increases subG0/G1	Increases caspase-3/7 activity and DNA fragmentation	[93]
	T24 RT4	Curcumin	15 15	G2/M	Decreases Trop2 and cyclin E1, increases p27	[94]
Glioma	U87MG	Quercetin	62.04	S	Inhibits ROS formation and angiogenesis	[95,96]
	LN229 GBM8401	Curcumin	5.85 6.31	G2/M	Increases apoptosis and ROS generation	[97]
	LN229 GBM8401	Dimethoxy curcumin	18.99 16.82	G2/M	Reduces p-mTOR, p-CDC2, and BCL-2, and increases p-AKT, p-ERK, LC3B-II, and p62	[97]
Leukemia	Nalm6	Quercetin	20	S	Intercalates into DNA, induces apoptosis by activating mitochondrial intrinsic pathway	[98]
	HL-60 ML-2 MOLM-13 OCI-AML3 OCI-AML5 U937	Curcumin	46.98 21.51 53.18 71.43 38.45 59.80	G1	Suppresses the phosphorylation of AKT, PRAS40, 4E-BP1, P70S6K, RAF-1, and p27 in ML-2 and OCI-AML5 cells	[99]

## Data Availability

Not applicable.
